# Electrical Treatment of Abdominal Viscera

**Published:** 1902-08-23

**Authors:** 


### ELECTRICAL TREATMENT OF ABDOMINAL VISCERA.

It has often been questioned what happens when attempts are made to treat the abdominal viscera by means of electricity. Which way does the current go? does it ever get into the muscular tissue of the stomach or intestines?—for it is as an aid to muscular contraction that electrical treatment is mostly used—and does it ever produce a normal peristalsis? With the object of throwing some light upon these questions Dr. McCaskey[1] has lately conducted a series of experiments which show pretty clearly that the passage of electricity, either in the galvanic or the faradic form, follows the ordinary physical laws, the current in every case passing by the lines of least resistance and diffusing over a considerable area between the electrodes. There seem to be practically three ways in which electricity may be used to stimulate the stomach and intestines:—(1) From skin to skin, the poles being so arranged as to include any desired viscus in the direct route between them. This is evidently the simplest although not the most effectual plan; (2) from mucous membrane to skin; (3) from the mucous membrane of one part, as for example of the stomach, to that of another, as of the colon. But in any case the current becomes widely diffused in the abdominal cavity, irrespective of the delimitations of the contained viscera. Faradism is the preferable form of electric treatment, and the technique of passing it from stomach to intestines is not difficult. An electrode is passed into the stomach, which should contain fluid, and the patient then lies down upon the operating table and a second electrode is passed into the colon. The current is then passed for about ten or fifteen minutes. If galvanism be used, care must be taken to have fluid in the bowel as well as in the stomach. What the passages of electricity from skin to skin does is probably to increase muscular tonus rather than to set up normal peristalsis, and this increase of tonus, when repeated, may produce permanent good effects. But when the current is passed from stomach to colon an active intestinal peristalsis is produced. It is impossible, however, to produce a physiological peristaltic wave of the stomach by any method of electrical stimulation, either experimentally or clinically.

[1] New York Med. Rec., July 26.

